# *Streptococcus Pyogenes* and Acute Rheumatic Fever: How Strong are the Links in the Chain?

**DOI:** 10.5334/gh.1564

**Published:** 2026-06-19

**Authors:** Scott H. Wirth, Andrea Z. Beaton, Andrew Steer

**Affiliations:** 1 Heart Center, Primary Children’s Hospital, Salt Lake City, Utah, USA; 2 Department of Pediatrics, University of Utah School of Medicine, Salt Lake City, Utah, USA; 3 Heart Institute, Cincinnati Children’s Hospital Medical Center, Cincinnati, Ohio, USA; 4 Department of Pediatrics, University of Cincinnati School of Medicine, Cincinnati, Ohio, USA; 5 Murdoch Children’s Research Institute, Royal Children’s Hospital, Flemington Road, Parkville, Victoria 3052, Australia

**Keywords:** Rheumatic Heart Disease, streptococcal pyogenes, vaccine licensure, primary prevention, historical review, narrative review

## Abstract

This narrative review synthesizes nearly two centuries of evidence linking *Streptococcus pyogenes* infection to acute rheumatic fever (ARF), the prerequisite for the development of rheumatic heart disease (RHD). Using the Bradford Hill criteria for causality and the Oxford Centre for Evidence-Based Medicine levels of evidence, we critically evaluate historical and contemporary data pertaining to this relationship. Strong evidence demonstrates that untreated *S. pyogenes* infection increases ARF incidence, while antibiotic treatment reduces incidence across diverse populations and settings. Moderate evidence supports the temporal sequence from infection to ARF and the exclusivity of *S. pyogenes* as ARF’s causal precursor. Weaker but biologically coherent data suggest an underlying mechanism consistent with post-infectious autoimmune disease. Although foundational evidence is robust, key gaps remain, particularly the need for contemporary prospective data in high-risk populations and clarity on the contribution of skin infections. Strengthening this evidence base is essential as *S. pyogenes* vaccines advance toward licensure and ARF-specific policy indications.

## Graphical Abstract

**Figure:**
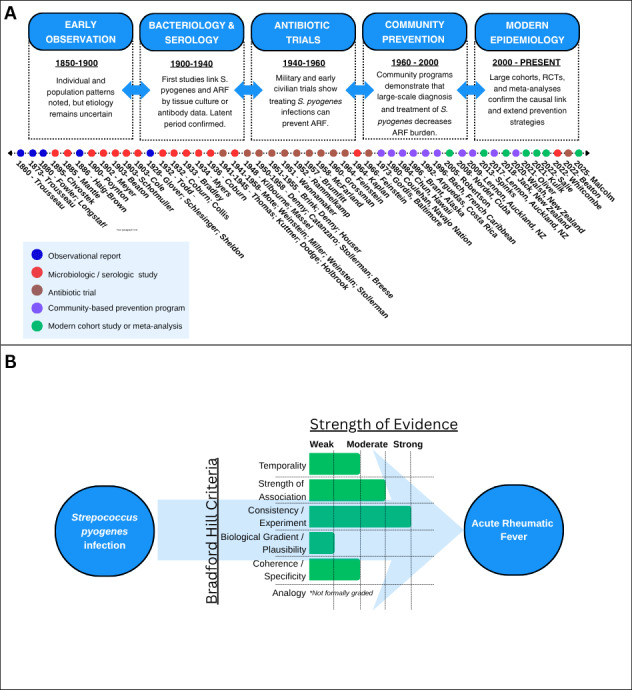

Synthesis of causal evidence linking *Streptococcus pyogenes* infection to acute rheumatic fever.

## Introduction

Rheumatic heart disease (RHD) affects >55 million people and causes >350,000 deaths annually, primarily in low-middle-income settings (1,2,3). Its burden among children and young adults makes it a major contributor to premature death, disability, healthcare costs, and reduced social and economic participation (4,5,6). RHD results from severe or recurrent episodes of acute rheumatic fever (ARF), an immune-mediated condition that can follow *Streptococcus pyogenes* infection of the pharynx or skin (3). This sequence underpins primary, secondary, and tertiary prevention strategies (7,8,9,10).

An effective *S. pyogenes* vaccine could markedly reduce the global burden of ARF and its sequel RHD. As multiple vaccine candidates enter phase III trials, an essential consideration is whether demonstrating prevention of *S. pyogenes* alone would justify a vaccine also having an indication for ARF prevention. Establishing the robustness of the causal linkage between *S. pyogenes* and ARF would strengthen the evidentiary basis for such licensure (11). To address this knowledge gap, we conducted a structured narrative review of historical and contemporary evidence linking *S. pyogenes* to ARF using two conceptual frameworks for evaluating causality and evidentiary strength.

## Methods

Literature pertaining specifically to the association between *S. pyogenes* and ARF was collected through a search of online and print-text databases and sources. Searches were conducted in PubMed, Google Scholar, and SCOPUS from 1800-April 20242 using combinations of keywords related to *S. pyogenes*, ARF, RHD, streptococcal infection, and prevention. Example search strings included ‘Streptococcus pyogenes’ AND ‘acute rheumatic fever’, ‘streptococcal infection’ AND rheumatic fever, and ‘group A streptococcus’ AND ARF AND prevention. English-language works were prioritized, with select French and German sources transliterated. Targeted review of authoritative print textbooks and historical monographs was also performed (12,13,14). These print sources provided primary historical context and extensive bibliographies that were used to identify additional original studies and reports, particularly from the 19^th^ and early 20^th^ centuries, that might not have been comprehensively indexed within these online databases.

Titles and abstracts were initially screened for relevance to the subject matter before full-text review. Studies were included if they provided observational or experimental data relevant to the epidemiology, natural history, risk factors, immunological basis, prevention, or treatment of *S. pyogenes* infection and/or ARF. There were no restrictions placed on size, population, study design, or statistical methodology. Studies pertaining primarily to the progression from ARF to RHD, the second step in the disease pathway, as well as other downstream outcomes were excluded from analysis, as were studies conducted in nonhuman systems.

Data extraction and synthesis were qualitative and narrative in nature, reflecting the heterogeneity and historical breadth of the included literature. For each study, relevant information was identified through structured review of study objectives, populations, exposure definitions, outcome measures, and key findings, with particular attention to elements pertinent to causal inference. Information was extracted with the specific aim of informing the structured frameworks we would later employ for analysis, rather than producing standardized quantitative summaries. However, key findings and figures, when presented quantitatively, were recorded. Where reporting formats or definitions varied, particularly in older studies, findings were interpreted in their historical and methodological context and compared conceptually rather than treated as directly equivalent measures.

The Bradford Hill criteria for causality and the Oxford Centre for Evidence-Based Medicine (OCEBM) levels of evidence, two distinct frameworks for the evaluation of scientific literature, were used sequentially and for complementary purposes to organize and appraise the collected data (15,16). To our knowledge, this is the first instance in which these two frameworks have been utilized in tandem for analysis in a historical or narrative review. This approach was selected because it allowed for a large collection of heterogeneous evidence to be organized and interpreted within structured, evidence-based frameworks while prioritizing transparency regarding study design, analysis, and evaluation of evidentiary rigor.

**Figure 1:**
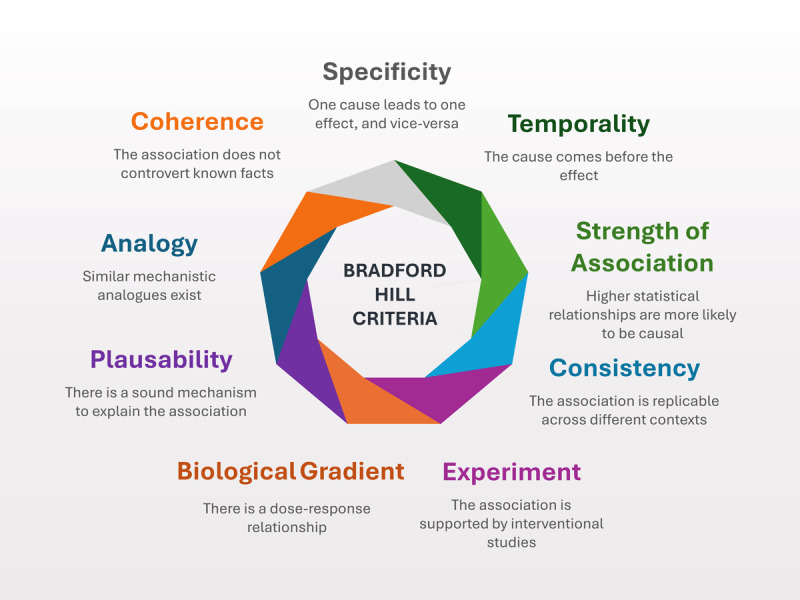
Bradford Hill criteria for causal inference—strength, consistency, specificity, temporality, biological gradient, plausibility, coherence, experiment, and analogy—used to evaluate the likelihood of a causal relationship.

**Figure 2:**
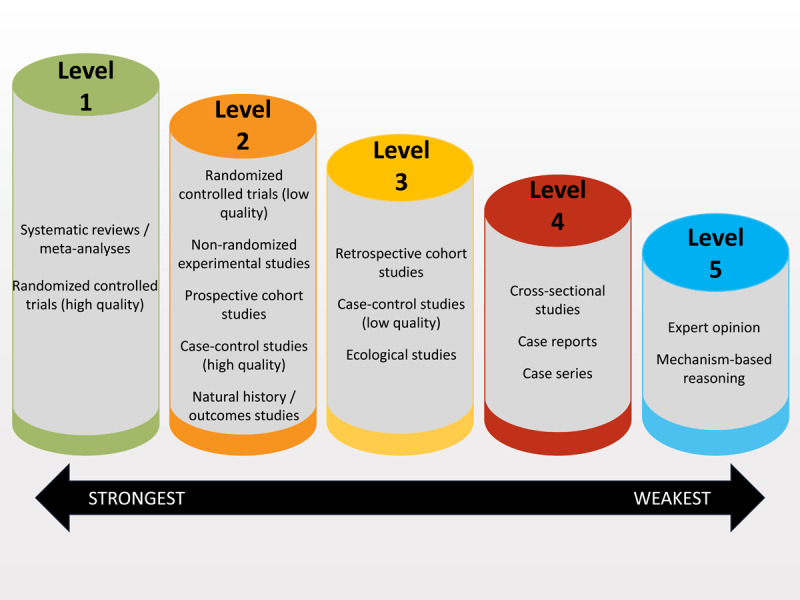
Modified Oxford Center for Evidence Based Medicine levels of evidence demonstrating the tiered hierarchy adapted for evaluating the strength of evidence included within this structured narrative review.

The Bradford Hill criteria (Figure 1) are a set of epidemiological considerations proposed to assess whether an observed association is likely to reflect a causal relationship rather than a mere correlation. Importantly, they do not form a rigid, all-or-nothing checklist. We used these criteria as an organizing framework, under which collected data were categorized according to the most pertinent fit. Assignment of studies to individual Bradford Hill criteria was performed using a consistent, structured interpretive approach. Each study was evaluated based on its primary aims, exposure and outcome definitions, and principal conclusions and categorized according to the causal consideration (i.e., Bradford Hill criterion) it most directly addressed. Where studies were relevant to multiple criteria, they were assigned based on their dominant contribution to causal inference, with secondary relevance acknowledged narratively. This process emphasized internal consistency and conceptual alignment rather than rigid or exclusive classification, in keeping with the non-prescriptive nature of the Bradford Hill criteria. Following categorization, the OCEBM levels of evidence (Figure 2) were used to appraise the collective methodological strength of research grouped under each Bradford Hill criterion. Evidence within each category was evaluated qualitatively, and an overall OCEBM level was assigned based on the most methodologically rigorous study or studies contributing to that criterion. This approach was chosen to reflect the strongest available evidence addressing each causal consideration while avoiding dilution by lower-quality or less directly informative studies.

Generative AI was used for editing and clarity. All scientific content, interpretation, and conclusions were developed and verified solely by the authors.

## Historical Setting

Accounts of ‘rheumatisms’ exist in the written record dating to ancient Greece and Rome, where early healers like Hippocrates and Celsus described constellations of joint pain, swelling, malaise, and weakness that occurred in children shortly following episodes of rhinorrhea (17,18,19,20). Modern reports, however, originate in the 17^th^ century. Writing in 1666, Thomas Sydenham made early efforts to divide febrile from non-febrile rheumatism, noting that the former often affected young people during wintertime and was distinguished by more severe joint pain, swelling, and redness (21).

By the mid-19th century, empirical observation began to replace archaic beliefs attributing illness to divine malfeasance and miasmic vapors floating through the air, a change that was heralded by John Snow’s advent of modern epidemiological methods during the 1854 London Cholera Outbreak (22). This paradigm shift prompted observations that ARF frequently followed sore throat and fever, often in populations also affected by scarlet fever (23,24,25,26). Still, ARF’s underlying mechanism remained unknown. As late as 1885, William Osler conceded that, ‘in rheumatic fever, we are still far away from any accurate knowledge of its intimate pathology’ (27).

Germ theory offered a path forward. By century’s end, growing recognition of the role of infectious microorganisms in human disease led to new theories on the pathogenesis of ARF (28,29,30). However, imprecise early microbiological techniques led to confused and often contradictory findings. Due to rudimentary culture techniques and an underappreciation of the risk of contamination, the period from 1900–1930 saw more than a dozen different bacteria implicated as the cause of ARF, sometimes many at once (31,32,33,34,35,36,37,38,39,40,41,42). Breakthrough finally arrived in 1931 when Coburn and Collis undertook simultaneous surveillance studies on their respective ARF wards. By sampling both asymptomatic and symptomatic individuals at regular weekly intervals, they found that *S. pyogenes* predominated in oropharyngeal cultures in the weeks prior to ARF, implicating the bacterium and describing the characteristic latent interval between infection and subsequent onset of rheumatic disease (43,44,45).

World War II accelerated progress (46). Crowded military bases, ripe with the spread of *S. pyogenes*, provided ideal conditions for large trials. The U.S. Streptococcal Disease Laboratory, established in 1948 at Warren Air Force Base in Wyoming, USA, produced pivotal evidence linking *S. pyogenes* to ARF and pioneered several antibiotic prophylaxis strategies (47,48,49,50,51,52,53,54,55). These findings would eventually shape global prevention programs for decades to come and remain foundational to current guidelines (56,57,58,59,60,61,62,63,64,65,66,67).

This historical trajectory from early clinical observations to rigorous bacteriologic and interventional studies cemented *S. pyogenes* as the causal agent of ARF and informed modern prevention strategies.

## Results

### Temporality: Does *S. pyogenes* precede ARF?

Temporality asks whether *S. pyogenes* infection precedes ARF and is among the most highly weighted Bradford Hill criteria.

#### Observational evidence

Late-19^th^-century reports noted ARF often followed exudative tonsillitis and shared seasonal overlap with scarlet fever and erysipelas (24,26). By the 1920s, these observations led to a proposed ‘latent period’ between infection and subsequent ARF onset, which was later quantified by Glover, Schlesinger, and Sheldon, each estimating ARF to occur 2–3 weeks following sore throat (39,42,68). Data from larger mid-century investigations corroborated their findings. In a 1963 study, 87% of ARF cases at La Rabida Sanitorium outside Chicago were preceded by a sore throat within the prior 5 weeks, while a 1969 secondary review of the Maryland ARF registry reported that 68% of ARF cases had similar antecedent pharyngitis (69,70).

#### Microbiologic evidence

Contemporaneous microbiological investigation, however, overlooked this latent period theory. They instead treated ARF as a primary infectious process rather than the aseptic sequelae of an infection. By choosing to sample fluid during an ARF episode, relying on rudimentary culture techniques, and not recognizing the problem of contamination, they produced widely heterogenous results that implicated a wide variety of bacteria, sometimes many at the time time, as the cause of ARF (31,32,33,34,35,71).

It was not until 1931 that Coburn and Collis sought to answer these questions through prospective microbiological surveillance studies. Drawing on their own clinical observations and these earlier case reports that had documented the temporal sequence from sore throat to acute rheumatic fever (ARF), they each designed prospective cohort studies in New York and London (23,24,25,26). Using weekly throat-culture surveillance of all staff and patients on their respective ARF wards, regardless of symptoms, they observed that individuals who developed sore throat consistently showed *Streptococcus pyogenes*–predominant throat flora and subsequently developed ARF approximately 2–3 weeks later. In contrast, asymptomatic participants maintained mixed bacterial flora and experienced no incident ARF (43,44). One year later, Bradley replicated these findings using an unrelated population of non-hospitalized British boarding school students (72). Similar patterns would be demonstrated in a much larger retrospective cohort study of U.S. Air Force recruits conducted after World War II, in which nearly half of recruits diagnosed with ARF had a preceding *S. pyogenes* throat culture documented within the preceding few weeks (73).

#### Immunological evidence

As a post-infectious, aseptic autoimmune condition, *S. pyogenes* is rarely recovered during ARF. However, elevated or rising streptococcal antibodies comprise one component of ARF’s diagnostic criteria (74). Their inclusion is derived from evidence dating to 1932, when Todd, using sera obtained from Coburn’s New York cohort, showed that participants with ARF had elevated anti-streptolysin-O compared to participants without ARF. His work established the immunological correlate to the latent period found in clinical and microbiological studies and has since been corroborated using a broad spectrum of immunoassays (75,76,77,78,79,80,81,82,83,84).

#### Emerging data on skin infections

Emerging evidence from Australia, New Zealand, and the Pacific Islands suggests that *S. pyogenes* skin infections may also trigger ARF (85,86,87). Case series have shown that the temporal relationship between impetigo and ARF mirrors that seen in pharyngitis, especially among Indigenous and underserved populations (85,88,89,90,91), though more research is needed to replicate this on a larger scale (92).

#### OCEBM Score

We graded the temporality criterion as OCEBM Level 3. Based on observational, microbiological, and immunological cohort and case-control studies that spanned diverse settings, *S. pyogenes* infection was consistently shown to precede ARF onset. However, study quality was limited either by retrospective design or small sample size.

### Strength of association: How strongly is *S. pyogenes* linked to ARF?

Strength of association quantifies the statistical relationship between *S. pyogenes* infection and ARF in terms of relative risk (RR) or odds ratio (OR). Here, we summarize and grade evidence from observational studies only.

#### Limited foundations

The mid-1900s were marked by several important large-scale observational studies into this question due to the establishment of the Streptococcal Research Laboratory at Warren Air Force Base in Wyoming. Here, researchers benefited from access to large populations of at-risk individuals, enabling larger cohort studies in settings where exposures and outcomes could be closely monitored. This shift represented a critical methodological advance and laid much of the groundwork for modern understanding of the *S. pyogenes*–ARF relationship. Yet even these studies remained constrained by rudimentary design and limited statistical analysis.

For example, Rammelkamp’s 1952 study of U.S. Air Force recruits was a single-group retrospective cohort of individuals with positive *S. pyogenes* throat cultures, reporting that 3% later developed ARF; without a comparator group, no potential difference from culture-negative controls could be evaluated (83). Contemporaneous controlled studies typically relied on simple comparisons of crude rates. Roughly a decade later, Wannamaker and Houser prospectively followed culture-positive and culture-negative recruits in similar U.S. Air Force settings and observed higher crude ARF rates among culture-positive individuals (2.7% vs 1.1%; 2.4% vs 1.4%) (93,94). However, subsequent reanalysis showed that these differences were not statistically significant (OR [95% CI] 1.48 [0.78, 2.81] and 1.74 [0.81, 3.73], respectively) (95). Issues such as these plague many of the investigations that came out of this research laboratory over its initial two-decade history.

#### Modern meta-analyses and large-scale cohort data

Modern analytical methods have enabled more rigorous evaluation of historical data. A 2021 meta-analysis incorporating the Wannamaker and Houser cohorts alongside a study of 110 Kyrgyzstani children found that individuals with a positive *S. pyogenes* throat culture had 1.74-times higher odds of developing ARF than those with a negative culture (95% CI 1.13–2.69) (95). This association is reinforced by recent population-scale evidence: in a retrospective analysis of over 1.8 million throat and skin cultures from Auckland, New Zealand, *S. pyogenes*–positive individuals had a relative risk of ARF of 4.8 (95% CI 3.6–6.4) for throat isolates and 5.1 (95% CI 1.8–15.0) for skin isolates compared with culture-negative participants (62).

#### OCEBM Score

We assigned an OCEBM Level 2 grade to this criterion. Although much of the literature remains limited by methodological constraints, the large New Zealand study provides highly compelling evidence linking *S. pyogenes* with ARF, albeit with potential biases inherent to its retrospective design. Future prospective studies using rigorous, contemporary methodologies would strengthen this criterion.

### Consistency and experiment: Can intervention prevent ARF?

The consistency and experiment criteria evaluate the stability of the exposure-outcome relationship. Consistency examines reproducibility across settings; experiment tests whether modifying the exposure alters the outcome. We consider them jointly here, focusing on interventional evidence.

#### Antibiotic trials in military and civilian populations

Interventional trials for ARF prevention emerged in the post-World War II era, enabled by the introduction of newly available antibiotics. By the late 1940s, researchers at the Streptococcal Disease Laboratory had initiated a series of pivotal trials evaluating sulfonamides, penicillin, and tetracyclines for both primary and secondary prevention of ARF (83,93,94,96,97,98,99,100,101,102,103). These studies generally demonstrated a protective effect against incident and recurrent ARF, though, like before, their methodological rigor was limited by the study designs of the time. Similar investigations soon extended to U.S. civilian populations, including schoolchildren in New York and Chicago, in whom penicillin similarly reduced ARF risk (104,105).

Collectively, these large interventional trials provided critical experimental evidence that antibiotic treatment or prophylaxis for *S. pyogenes* infection substantially reduces the risk of ARF. Subsequent meta-analyses of military and civilian studies found that primary antibiotic prophylaxis lowered ARF risk compared with no treatment (RR = 0.32; 95% CI 0.21–0.46), with an analysis focused specifically on penicillin demonstrating an even greater protective effect (RR = 0.20; 95% CI 0.11–0.36) (106,107). A summary of experimental antibiotic studies can be found in Table 1.

**Table 1:** Summary of historical antibiotic trials evaluating whether antibiotic treatment of *Streptococcus pyogenes* tonsillopharyngitis reduces subsequent acute rheumatic fever.


STUDY	DESIGN	POPULATION	INTERVENTION	ENDPOINT	RESULTS	CONCLUSIONS

Rammelkamp 1952 (81)	Retrospective analysis of hospital records	U.S. Air Force servicemen with *S. pyogenes*-positive tonsillopharyngitis, n = 1,974	Penicillin of varying doses and schedules vs no treatment	ARF incidence within 34 days of symptom onset	ARF developed in 1/978 in treatment arm vs 23/996 in control arm	The rate of ARF was 23× higher in untreated controls

Wannamaker 1951 (91)	Multi-arm randomized controlled trial (RCT)	U.S. Air Force servicemen with sore throat (no use of cultures to verify *S. pyogenes)*, n = 2,340	IM Penicillin vs no treatment: - Arm 1: 300,000 units on day 1, 300,000 units on day 2, 600,000 units on day 4 - Arm 2: 300,000 units day 1, 300,000 units day 3 - Arm 3: 600,000 units day 1	ARF incidence within 45 days of symptom onset	ARF developed in: - Arm 1: 3/516 treatment, 22/487 control - Arm 2: 1/200 treatment, 8/239 control - Arm 3: 1/262 treatment, 5/270 control - Total: 5/578 treatment, 35/996 control	The rate of ARF was ~7× higher in untreated controls

Houser 1953 (92)	Multi-arm RCT	Hospitalized U.S. Air Force servicemen with exudative tonsillopharyngitis (no use of cultures to verify *S. pyogenes*), n = 2,044	Oral Aureomycin vs no treatment: - Arm 1: 0.1 g at enrollment, then 0.5 g every 4 hours × 5 doses, then 0.25 g every 4 hours × 30 doses - Arm 2: 0.1 g at enrollment, then 0.5 g every 6 hours × 19 doses - Arm 3: 1.0 g at enrollment, then 0.5 g every 4 hours × 5 doses, then 0.25 g every 4 hours × 18 doses	ARF incidence within 21 days of symptom onset	ARF developed in: - Arm 1: 1/108 treatment, 1/112 control - Arm 2: 15/622 treatment, 24/624 control - Arm 3: 4/279 treatment, 5/299 control - Total: 20/1009 treatment, 29/1034 control	The rate of ARF was ~1.5× higher in untreated controls

Denny 1953 (94)	Multi-arm RCT	Hospitalized U.S. Air Force servicemen with *S. pyogenes* positive exudative tonsillopharyngitis, n = 207	IM Penicillin vs Aureomycin vs Oral Terramycin vs placebo - Arm 1: Penicillin 600,000 units once daily × 5 days - Arm 2: Aureomycin 1 g at enrollment, then 0.5 g every 6 hours × 19 doses - Arm 3: Terramycin 1 g at enrollment, then 0.5 g every 6 hours × 19 doses - Arm 4: Placebo	ARF incidence	ARF developed in total of 3/207 participants across all study arms.	The rate of ARF was too low to calculate differences among study arms

Brink 1951 (95)	Multi-arm RCT	Hospitalized U.S. Air Force servicemen with exudative tonsillopharyngitis, (no use of cultures to verify *S. pyogenes)* n = 475	IM Penicillin vs Oral Aureomycin vs no treatment - Arm 1: Penicillin 300,000 units at enrollment, then 300,000 units at 48 hours, then 600,000 units at 96 hours - Arm 2: Aureomycin 1 g at enrollment, then 0.5 g every 4 hours × 6 doses, then 0.25 g every four hours × 18 doses - Arm 3: No treatment	ARF incidence within 10–35 days of symptom onset	ARF developed in - Arm 1: 2/197 - Arm 2: 0/80 - Arm 3: 5/198	The rate of ARF was 2.5× higher in untreated controls

Catanzaro 1955 (96)	Multi-arm RCT	Hospitalized U.S. Air Force servicemen with exudative tonsillopharyngitis, (no use of cultures to verify *S. pyogenes)* n = 986	Oral Oxytetracycline vs no treatment - Arm 1: 1.0 g at enrollment, then 0.5 g every 6 hours × 19 doses - Arm 2: 0.5 g four times daily × 20 doses - Arm 3: 0.5 g every 6 hours × 20 doses	ARF incidence	ARF developed in 12/506 in combined treatment groups and 19/480 in combined control groups.	ARF developed in 12/506 in combined treatment groups and 19/480 in combined control groups.

Denny 1950 (97)	RCT	Hospitalized U.S. Air Force servicemen with exudative tonsillopharyngitis, (no use of cultures to verify *S. pyogenes)* n = 1,634	IM Penicillin vs no treatment - 300,000 units at enrollment, then 300,000 units at 48 hours, then 600,000 units at 96 hours	ARF incidence between 21–28 days following symptom onset	ARF developed in 4/798 in treatment group and 23/804 in control groups.	The rate of ARF was ~5.5× higher in untreated controls

Catanzaro 1954 (98)	RCT	Hospitalized U.S. Air Force servicemen with exudative tonsillopharyngitis, (no use of cultures to verify *S. pyogenes)* n = 1,177	IM Penicillin vs no treatment – 900,000 units on day 9, 11, and 13 after illness	ARF incidence within 45 days following symptom onset	ARF developed in 3/219 in treatment group and 11/220 in control group.	The rate of ARF was ~3.5× higher in untreated controls

Siegel 1961 (102)	Nonrandomized prospective trial	U.S. children at outpatient clinic in Chicago, Illinois, with *S. pyogenes* tonsillopharyngitis, n = 1,213	IM Penicillin vs no treatment – 900,000 units ×1 dose	ARF incidence	ARF developed in 0/605 in treatment group, 2/608 in control group.	The rate of ARF was too low to calculate differences among study arms

Breese 1953 (103)	Multi-arm, nonrandomized prospective trial	U.S. children at outpatient clinic in Rochester, New York, with *S. pyogenes* tonsillopharyngitis, n = 792	IM or oral penicillin vs aureomycin vs sulfadiazine vs no treatment - Arm 1: IM penicillin or oral penicillin of variable doses and schedules - Arm 2: Aureomycin 10mg/lb once daily × 2 days, then 5mg/lb once daily × 8–12 days - Arm 3: Sulfadiazine 0.06g/lb once daily × 5–8 days	ARF incidence	ARF developed in total of 1/792 participants across all study arms.	The rate of ARF was too low to calculate differences among study arms

Robertson 2005 (104)	Meta-analysis	10 hospital-based studies, 8/10 of which occurred on U.S. military bases. 2/10 included children. All studies limited inclusion to subjects with exudative pharyngitis, but few used culture-based diagnostic criteria, n = 3996 participants	Antibiotics (inclusive of IM penicillin, oral Aureomycin, oral Terramycin) versus control (placebo or no treatment)	ARF incidence	For all antibiotics, ARF developed in 29/3996 in treatment group, 89/3669 in control groups. Pooled RR 0.32 (95% CI 0.21–0.48). For penicillin only, ARF developed in 12/3464 in treatment group, 63/3238 in control groups. Pooled RR 0.20 (0.11–0.36).	The rate of ARF was ~3× higher in untreated controls compared to all antibiotics, and ~4× higher in untreated controls compared to penicillin alone.

Spinks 2013 (105)	Meta-analysis	16 studies included in analysis of all antibiotics for treatment of sore throat, 14 included in penicillin-only sub-analysis. 8 studies include only U.S. Air Force recruits in 1950s, remainder included mix of children and adults.	Antibiotics vs control (placebo or no treatment)	ARF incidence	For all antibiotics, ARF developed in 37/5656 in treatment group, 124/4445 in control groups. Pooled RR 0.27 (0.12–0.60) For penicillin only, ARF developed in 21/4332 in treatment group, 74/3843 in control groups. Pooled RR 0.27 (0.14–0.50)	The rate of ARF was ~3.7× higher in untreated controls compared to all antibiotics and compared to penicillin alone.


This table synthesizes key interventional and observational studies from military and civilian settings assessing whether antibiotic treatment of *S. pyogenes* pharyngitis reduces subsequent ARF risk. It details study design, populations, treatment regimens, endpoints, and reported outcomes, illustrating how early and modern evidence contributed to establishing infection treatment as a cornerstone of ARF prevention.

#### Extrapolation to community prevention programs

These trials helped catalyze widespread primary prevention programs in RHD-endemic regions worldwide, including inner-city Baltimore (57), the French Caribbean (59), the Navajo Nation (60), New Zealand (64,65,66,67), and rural communities in Cuba (58), Hawaii (61), Alaska (62), and Costa Rica (63). Although all sought to expand access to primary antibiotic prophylaxis, their surveillance strategies differed. Some, such as those in New Zealand, offered school-based sore throat monitoring using weekly throat cultures (64,65,66,67). Others, like those in Alaska, Cuba, and Costa Rica, emphasized community-based surveillance, enacting widespread ARF educational campaigns meant to increase awareness and referral within the existing public health infrastructure (58,62,63). Each reported reductions in ARF incidence, though study designs ranged from quasi-experimental before-and-after approaches to randomized or cluster-randomized trials. A subsequent meta-analysis incorporating these data found significant reductions in ARF among individuals receiving primary antibiotic prophylaxis (RR 0.41; 95% CI 0.20–0.70), further reinforcing the causal relationship between *S. pyogenes* and ARF (67,108). A summary of primary prevention programs can be found in Table 2.

**Table 2:** Summary of community- and school-based primary prevention programs aiming to reduce acute rheumatic fever incidence through early detection and treatment of *Streptococcus pyogenes* infections.


STUDY	DESIGN	SETTING	POPULATION	INTERVENTION	RESULTS

Gordis 1973 (56)	Comparison of before and After intervention (Pre/Post)	Baltimore, Maryland, USA 1960–1980	Clinic-based children ages 5–14 years old, primarily low-income African American, with clinical diagnosis of *S. pyogenes* tonsillopharyngitis (no use of cultures to verify *S. pyogenes)*.	Establishment of comprehensive general primary care clinics in neighborhoods with high incidence of ARF	60% reduction in ARF incidence

Nordet 2008 (57)	Pre/Post	Pinar del Rio Province, Cuba 1986–1996	Community-, clinic-, and hospital-based children age 5–25 years old with permanent resident in province, with culture-based diagnosis of *S. pyogenes* tonsillopharyngitis.	Public health awareness campaign, training of health personnel, epidemiologic sore throat surveillance with culture-based case confirmation, antibiotics	Decrease in incidence of first ARF from 12·2/100,000 to 2·1 per 100,000 among all ages. In children ages 5–14 years old, incidence decreased from 23·4/100,000 to 1·8/100,000.

Bach 1996 (58)	Pre/Post	Martinique and Guadeloupe 1981–1991	Community-, clinic-, and hospital-based children ages 5–18 years old living in Martinique or Guadeloupe, with culture-based diagnosis of *S. pyogenes* tonsillopharyngitis.	Public health awareness campaign, epidemiological sore throat surveillance with culture-based case confirmation, antibiotics	78% reduction in ARF incidence in Martinique, 74% reduction in Guadeloupe

Coulehan 1980 (59)	Cluster RCT	Navajo Nation 1962–1977	School-based children in Navajo Native American Tribe, with culture-based diagnosis of *S. pyogenes* tonsillopharyngitis.	School-based sore throat clinic with culture-based case confirmation, nurse-observed antibiotics	39% reduction in ARF incidence in intervention group compared to control group

Chun 1984 (60)	Retrospective cohort study	Oahu, Hawaii 1976–1980	Children hospitalized with ARF in Oahu, with culture-based diagnosis of *S. pyogenes* tonsillopharyngitis.	School-based sore throat clinic with culture-based case confirmation, nurse-observed antibiotics	No difference in ARF incidence among children in a school with program vs those in a school without program

Brant 1986 (61)	Pre/Post	Alaska 1971–1976	Community-based Alaskan Eskimo children, with culture-based diagnosis of *S. pyogenes* tonsillopharyngitis.	Community-based sore throat clinic with culture-based case confirmation, antibiotics	Decrease in incidence of ARF from 11/100,000 to 0/100,000 in communities with program.

Arguedas 1992 (62)	Pre/Post	Costa Rica 1950–1990	Clinic- and community-based children in Costa Rica, with clinical diagnosis of *S. pyogenes* tonsillopharyngitis (no use of cultures to verify *S. pyogenes*).	Public health awareness campaign, use of clinical diagnostic score rather than throat cultures for case confirmation, and adoption of only IM penicillin (rather than oral penicillin)	Decrease in incidence of first ARF from 120/100,000 (1950s) and 90/100,000 (1970s) to 7/100,000 (1985) and 1/100,000 in 1990. Decrease in annual ARF cases referred to national hospital from 94 (1970) to 4 (1991).

Jack 2018 (63)	Retrospective cohort study	New Zealand 2009–2016	School-based children in New Zealand with culture-based diagnosis of *S. pyogenes* tonsillopharyngitis.	Nationwide school-based sore throat clinic with culture-based case confirmation and nurse-monitored antibiotics	28% reduction in ARF incidence nationally 46% reduction in ARF incidence among high-risk populations.

Lennon 2017 (64)	Pre/Post	Auckland, New Zealand 2010–2016	School-based children in Auckland, New Zealand with culture-based diagnosis of *S. pyogenes* tonsillopharyngitis	City-wide school-based sore throat clinic with active culture surveillance and antibiotics	58% reduction in ARF incidence after 2 years

Walsh 2020 (65)	Pre/Post	New Zealand 2000–2018	School- and community-based Māori children in New Zealand with culture-based diagnosis of *S. pyogenes* tonsillopharyngitis	- Cohort 1- School-based sore throat clinic with culture-based case confirmation and antibiotics, General Practitioner (GP) support – Cohort 2- GP only care - Cohort 3- GP care plus limited coverage with school-based sore throat clinics	- Cohort 1: 60% reduction in ARF incidence - Cohort 2: nonsignificant 128% increase in ARF incidence - Cohort 3: 48% reduction in ARF incidence

Lennon 2009 (66)	Cluster RCT	Auckland, New Zealand 1998–2001	School-based children in Auckland, New Zealand with culture-based diagnosis of *S. pyogenes* tonsillopharyngitis	School-based sore throat clinic, nurse-observed antibiotics	Non-significant 21% reduction in ARF incidence


This table presents major public health interventions across diverse geographic and socioeconomic contexts, describing program design, target populations, diagnostic approaches, and ARF outcomes. Collectively, these programs demonstrate how systematic sore-throat surveillance and timely antibiotic treatment have impacted ARF incidence in real-world, population-level settings.

#### Secondary prevention and the GOAL trial

Compared with the well-established evidence for primary prophylaxis, experimental data on secondary prophylaxis—the long-term use of antibiotics to prevent ARF recurrence and progression to RHD—remains limited. Secondary prophylaxis often requires decades of continuous adherence (109), and adherence can be undermined by pain, stigma, transportation barriers, limited healthcare access, and other structural challenges (107,108,109,110,111,112,113,114). The long latency between ARF and subsequent RHD further complicates research, increasing cost and vulnerability to loss of follow-up and failure of protocol. Despite these challenges, available observational data indicate that secondary prophylaxis is highly effective in preventing recurrent ARF and halting RHD progression (110,111,112). Until recently, however, robust experimental evidence was sparse.

The GOAL trial, published in 2022, addressed this gap. Conducted in Uganda, it enrolled 800 children with early, asymptomatic RHD into a randomized controlled trial evaluating monthly penicillin injections for preventing disease progression (113). Penicillin prophylaxis significantly reduced progression to more advanced RHD compared with controls, an effect that was presumed to result from prevention of recurrent *S. pyogenes* infections and ARF episodes. Given the demonstrated efficacy, newer strategies aimed at overcoming barriers to long-term adherence, such as continuous subcutaneous penicillin depots, are now under investigation (114).

#### OCEBM Score

We assigned an OCEBM Level 1 grade to the evidence supporting the consistency and experiment criteria. Although early trials were conducted in less generalizable populations and prevention programs varied in design, the volume and reproducibility of findings across settings justify a strong rating. Consistent reductions in ARF incidence following antibiotic treatment of *S. pyogenes* infections across both clinical trials and real-world programs provide compelling causal evidence.

### Biological gradient and plausibility: Is more exposure linked to greater risk?

The biological gradient and plausibility criteria assess whether a dose-response relationship exists between *S. pyogenes* and ARF, and whether a coherent physiological mechanism supports that relationship.

#### Evidence for a dose–response relationship

A simple, linear dose–response relationship between *S. pyogenes* and ARF has not been definitively established, but multiple lines of evidence suggest that a complex biological gradient that operates through repeated exposure and host susceptibility might exist.

Observational studies have long documented higher ARF incidence in crowded, unhygienic environments, such as military barracks and densely populated urban settings, where exposure to *S. pyogenes* is more frequent (92,115,116,117,118). These ecological associations suggest that increased opportunity for repeated infection may elevate ARF risk, although they do not establish a quantifiable, linear exposure-response relationship.

Immunologic data provide additional support for a biological gradient. As early as 1933, Schlesinger demonstrated that patients who go on to develop ARF exhibit higher antistreptococcal antibody responses than those with uncomplicated *S. pyogenes* infection, a finding that has been repeatedly confirmed in subsequent serologic studies using increasingly sophisticated assays (78,100,119,120,121,122,123). More recent investigations from New Zealand have extended these observations, showing that ARF patients also mount broader and more heterogeneous antibody responses than those who recover without sequelae (124,125,126).

These findings require cautious interpretation. Rather than demonstrating a simple, linear dose-response relationship, the substantial inter-individual heterogeneity in immune responses to *S. pyogenes* infection suggests a more complex relationship that is affected by host factors. Rather than merely higher antibody titers, patients with ARF exhibit qualitatively distinct immune profiles from each other and from non-ARF peers. Additionally, to date, no study has shown that immune responses scale proportionally with the severity or frequency of antecedent infection. These data instead suggest that immune priming and breadth of response, likely reflecting repeated exposure in susceptible hosts, are more strongly associated with ARF risk than exposure ‘dose’ alone.

#### Is the mechanism biologically plausible?

The pathway linking *S. pyogenes* infection to ARF and subsequent RHD is widely accepted but remains incompletely defined. The leading model proposes that anti-streptococcal antibodies cross-react with host tissues through molecular mimicry, inducing inflammation in the heart, joints, and nervous system, accounting for ARF’s multisystem presentation (127,128,129,130). Alternative models emphasize dysregulated cellular immunity, including aberrant T-cell activation, cytokine amplification, innate immune skewing, and loss of immune tolerance (131,132,133,134,135,136). Contemporary immunologic studies in ARF patients have documented Th1/Th17 polarization, B-cell activation, and complement activation, further supporting an immune-mediated mechanism (122,125,132,133,137,138). Despite these insights, the precise integration of these pathways and their relationship to *S. pyogenes* infection remain unresolved.

#### OCEBM Score

We assigned an OCEBM Level 4 grade to the evidence supporting this criterion. While indirect evidence suggests a biological gradient, definitive data linking *S. pyogenes* burden to ARF risk remain lacking, and the immunologic pathogenesis of ARF is still not fully delineated.

### Analogy: Are there comparable immune-mediated responses to infection?

The analogy criterion considers whether comparable causal relationships exist elsewhere in medicine, particularly those in which localized infections trigger systemic immune-mediated syndromes. Although generally viewed as the weakest of the Bradford Hill criteria, identifying other analogous mechanisms can strengthen the case for a causal link between *S. pyogenes* and ARF.

Several well-recognized analogs support this criterion. Guillain-Barré syndrome follows a molecular-mimicry pathway like that proposed for ARF, in which antibodies generated against *Campylobacter jejuni* cross-react with peripheral nerve gangliosides, causing demyelination and ascending paralysis (139). Reactive arthritis develops after gastrointestinal or genitourinary bacterial infection and involves aberrant immune activation leading to sterile joint inflammation—paralleling the migratory arthritis of ARF (140). Post-streptococcal glomerulonephritis, closely linked to *S. pyogenes*, occurs when immune complexes containing anti-streptococcal antibodies deposit in glomeruli, triggering complement-mediated renal injury (141). Outbreaks of glomerulonephritis and ARF have also been reported concurrently in regions such as Trinidad and Chile, where overlapping demographic and environmental risk factors suggest shared exposure and susceptibility (90,142,143,144).

#### OCEBM Score

We did not assign an OCEBM grade to this criterion. Although these analogies do not establish causality, they help support the conceptual framework linking *S. pyogenes* to ARF.

### Coherence and specificity: Is ARF only caused by *S. pyogenes*?

Coherence requires that a proposed causal relationship align with established biological understanding, while specificity considers whether an outcome is attributable to a single exposure. As with analogy, specificity is among the least influential Bradford Hill criteria, given that many diseases have multifactorial etiologies. Nonetheless, we examine these criteria together to assess whether any credible alternative triggers for ARF exist beyond *S. pyogenes* infection.

#### Early misconceptions

Early 20th-century theories frequently attributed ARF to pathogens other than *S. pyogenes*, sometimes suggesting a single alternative organism or a polymicrobial cause. These ideas stemmed from inconsistent microbiological findings: early culture techniques often isolated diverse bacteria, such as staphylococci, pneumococci, and diphtheroids, from patients during ARF episodes rather than during their antecedent infections, fueling speculation about a multifactorial infectious origin (31,32,33,34,35,71,141,145). Even after the pivotal work of Coburn and Collis, skepticism persisted. T. Duckett Jones, for example, questioned the specificity of *S. pyogenes* as the trigger because many ARF patients did not recall a preceding episode of tonsillopharyngitis (57,69,146). Although this is now understood to reflect subclinical *S. pyogenes* infections, Jones argued that ARF must be multifactorial, potentially arising from factors such as nutritional deficiency, environmental conditions, trauma, surgery, or even typhoid vaccination (147). Accordingly, the earliest versions of his diagnostic framework, the Jones Criteria, did not include any elements specific to *S. Pyogenes* (148).

#### Modern evidence

Subsequent literature strongly supports ARF’s exclusive association with *S. pyogenes*. In a 1945 U.S. study, 15 of 400 individuals with positive throat cultures developed ARF, whereas none of 1,100 culture-negative individuals did so (149). Similar results emerged from New Zealand’s Rheumatic Fever Prevention Programme, which screened hundreds of thousands of children through school-based sore-throat clinics from 2012 to 2016 and documented zero cases of ARF among children with negative cultures (64).

#### OCEBM Score

Although specificity is considered a weaker Bradford Hill criterion, findings across multiple large datasets are remarkably consistent. We identified no credible evidence implicating any trigger for ARF other than *S. pyogenes* infection. We assigned an OCEBM Level 3 grade to the coherence and specificity criteria, as the strongest supporting data derive from large-scale observational studies and public health surveillance. However, the relative absence of prospective interventional trials limits the strength of the evidence.

## Discussion

Over the past century, evidence linking *S. pyogenes* to ARF has advanced from early clinical observations to data obtained from controlled epidemiological studies, interventional experiments, and population-scale primary prevention programs. By applying the Bradford Hill criteria and OCEBM grading, we conclude that *S. pyogenes* is the sole and necessary precursor to ARF.

The strongest support for this assertion comes from strength of association (Level 2) and consistency/experiment (Level 1) criteria. Meta-analyses show that untreated infection substantially increases ARF risk, while antibiotic therapy reduces it by ~80%, with consistent effects demonstrated across diverse populations and settings.

Moderate support derives from temporality and coherence/specificity (Level 3) criteria, which demonstrate that ARF follows infection after a 2–3-week latent interval. Biological gradient and plausibility (Level 4) criteria provide weaker but still meaningful evidence; although mechanisms remain incompletely described, an immune-mediated model is biologically credible. The analogy criterion, though ungraded, reinforces plausibility through parallels with other post-infectious syndromes.

Taken together, evidence spanning OCEBM Levels 1–4 supports a strong causal link between *S. pyogenes* infection and ARF. This foundation is critical for vaccine development, confirming that preventing *S. pyogenes* infection should prevent ARF, the prerequisite for RHD.

Several limitations merit acknowledgement. This review was intentionally focused on the evidence linking *S. pyogenes* infection to ARF and excluded literature pertaining to the progression from ARF to RHD. This focused scope reflected current regulatory needs in the vaccine development pathway. Though mitigation of RHD ultimately underlies most prevention efforts, RHD’s chronic, progressive nature makes it an impractical research endpoint. ARF, conversely, occurs within weeks following *S. pyogenes* and represents a more operationally feasible outcome measure for vaccine trials. Our review focused on providing the evidentiary basis for its use as such. Second, much of the foundational literature predates modern indexing practices, and no single database comprehensively catalogs all 19^th^-century biomedical publications. Some historical reports remain accessible only through print or archival sources. To mitigate this, we supplemented web-based database searches with reviews of authoritative historical textbooks and monographs and manually traced citations to ensure inclusion of seminal studies. Nonetheless, some historical gaps undoubtedly remain and should be considered in interpreting the early evidence. Third, many foundational studies underpinning the *S. pyogenes*-ARF relationship were conducted in U.S. military cohorts that are not fully generalizable to today’s highest-risk populations, lacked standardized case definitions, and utilized methodological designs that predate modern epidemiologic standards. To account for this problem, future research should prioritize prospective studies in endemic settings that incorporate contemporary diagnostic approaches, including standardized echocardiographic criteria where appropriate. Finally, additional investigation is also needed to clarify the contribution of skin infections to ARF pathogenesis and to improve long-term follow-up with robust cardiac outcomes.

## Conclusions

Historical data suggest that *S. pyogenes* is the definitive cause of ARF, providing a strong evidentiary basis for vaccine development. By systematically applying the Bradford Hill criteria and Oxford Centre for Evidence Based Medicine framework, we demonstrate that this relationship is supported by consistent epidemiologic, microbiologic, immunologic, and interventional data across diverse populations and settings.

This evidentiary foundation has important implications for prevention. Interventions that prevent or effectively treat *S. pyogenes* infection are expected to prevent ARF, which is the prerequisite for the development of RHD. Although progression to chronic cardiac disease was not the focus of this study, our findings underscore the potential downstream impact of interrupting ARF at its source.

Closing remaining evidence gaps will require contemporary prospective studies conducted in high-risk populations using standardized diagnostic criteria and long-term follow-up. Such efforts will be essential to meet regulatory standards and achieve meaningful reductions in the global burden of ARF and RHD.

## Summary

This structured review synthesizes two centuries of evidence linking *Streptococcus pyogenes* infection to acute rheumatic fever and rheumatic heart disease, critically assessing the robustness of this causal relationship and identifying key evidence gaps necessary to underpin future licensure of vaccines targeting these conditions.

## Data Availability

This study is a narrative review and does not involve generation of new datasets. All data supporting the findings are derived from previously published sources cited within the manuscript. No additional datasets were created or analyzed.
